# Investigating molecular interactions between oxidized neuroglobin and cytochrome c

**DOI:** 10.1038/s41598-018-28836-6

**Published:** 2018-07-12

**Authors:** Purushottam B. Tiwari, Prem P. Chapagain, Aykut Üren

**Affiliations:** 1 Department of Oncology, Georgetown University, Washington D.C., USA; 2 Department of Physics, Florida International University, Miami, FL USA; 3 Biomolecular Sciences Institute, Florida International University, Miami, FL USA

## Abstract

The formation of a complex between neuroglobin (Ngb) and cytochrome c (Cyt c) has an important biological role in preventing apoptosis. Binding of Ngb to Cyt c alone is sufficient to block the caspase 9 activation by ferric Cyt c that is released during ischemic insults. Therefore, a detailed information on the Ngb-Cyt c interactions is important for understanding apoptosis. However, the exact nature of the interactions between oxidized human neuroglobin (hNgb) and Cyt c is not well understood. In this work, we used a combination of computational modeling and surface plasmon resonance experiments to obtain and characterize the complex formation between oxidized hNgb and Cyt c. We identified important residues involved in the complex formation, including K72 in Cyt c, which is otherwise known to interact with the apoptotic protease-activation factor-1. Our computational results, together with an optimized structure of the hNgb-Cyt c complex, provide unique insights into how the hNgb-Cyt c complex can abate the apoptotic cascade without an hNgb-Cyt c redox reaction.

## Introduction

Neuroglobin (Ngb) is a six-coordinated heme protein that is mainly expressed in nervous and endocrine tissues^1^ and the retina^2^. The structure of Ngb is similar to that of myoglobin^3^. Ngb has been proposed to be involved in the regulation of Alzheimer’s disease^4^. During hypoxic-ischemic insults, Ngb remains up-regulated and protects neurons^5^. However, the mechanism of Ngb neuroprotection is unclear^6,7^, and further studies investigating the neuroprotective role of Ngb are required^8^. Therefore, recent studies have increasingly explored the binding partners of Ngb^9–12^.

Cytochrome c (Cyt c), which is another heme protein^13^, is a known Ngb binding partner^12,14^. This mitochondrial heme protein triggers cell apoptosis during apoptotic cell signaling^15^. During ischemic insults, the cellular apoptotic pathway is activated, leading to the release of ferric Cyt c, which is an essential component of the apoptosome, into the cytoplasm^16–18^. Since a redox reaction between Ngb and Cyt c is not required to prevent the formation of the apoptosome, the binding of Ngb to Cyt c alone is sufficient to block the Cyt c-induced caspase 9 activation^19^. Thus, investigations targeting these two heme proteins in their ferric state are important to the scientific community.

According to studies using surface affinity-based surface plasmon resonance (SPR), nanopore-based nanopipettes, and solution-based isothermal titration calorimetry (ITC) experimental techniques, ferric human Ngb (referred to as hNgb hereafter) interacts with ferric Cyt c (referred to as Cyt c hereafter) with a micromolar affinity^12,14,20^. Electrochemical impedance spectroscopy measurements have also confirmed the formation of an hNgb-Cyt c complex^12^. Computational docking is useful to investigate the complex formation between proteins^21^. Several computational docking studies have been performed to predict Ngb-Cyt c complexes^12,14,19^. These computational techniques complement the limitations of the experimental methods used to study protein-protein interactions (PPIs)^22^. Molecular docking is useful in predicting the binding interfaces of interacting partners^23,24^. Since proteins are flexible biomolecules, a dynamic reorganization in the structures of the interacting partners occurs during PPIs^12^, and these conformational dynamics are the fundamental basis for their structure and function^25^. Molecular dynamics (MD) simulations are useful in investigations exploring the mechanisms by which the conformational flexibility of a protein is utilized in complex formation and stabilization^26^. Therefore, MD simulations are routinely used to study complex formation between a protein and its interacting partners^21,27,28^ and are crucial for an understanding of the physical basis of biomolecular structure and function^25^.

While experimental and docking-based computational investigations have been performed, the optimized structure of this important hNgb-Cyt c protein-protein complex has not been established. Therefore, the exact nature of the hNgb-Cyt c complex is not well understood. Here, we investigated the formation and stabilization of the complex between hNgb and Cyt c. We first modeled the hNgb-Cyt c complex using molecular docking and then performed MD simulations of the docked complex. The complex is stable (does not dissociate) for 700 ns of the simulation time, despite major structural changes. We observed an interesting structural rearrangement of the complex during stabilization. Based on our results, we predict that hNgb interacts with Cyt c via two salt bridges between D73 (hNgb) and K72 (Cyt c) and between E87 (hNgb) and K27 (Cyt c) as well as a hydrogen bond between T77 (hNgb) and K72 (Cyt c). The involvement of the amino acid residues predicted by the MD simulations was confirmed by SPR. To the best of our knowledge, this is the first computational investigation of the molecular mechanism underlying the hNgb-Cyt c complex formation, allowing conformational flexibility and structural rearrangements.

## Results

### Two salt bridges and a hydrogen bond are responsible for the formation and stabilization of the hNgb-Cyt c complex

The best predicted hNgb-Cyt c complex (see Materials and Methods section) structure obtained from docking studies was used in MD simulations. In this best predicted structure, several interfacial atomic contacts initiated the formation of the complex between hNgb and Cyt c. The formation of a complex between protein biomolecules is a dynamic process involving the structural reorganization of the interacting partners^21^. Additionally, proteins are flexible biomolecules, and their structure and function depend on their conformational flexibility^25^. The optimization of interfacial contacts via the structural adjustment of interacting protein molecules can be realized by dynamics^21^, providing the residues with enhanced flexibility. Therefore, to examine the stability and optimize the conformational integrity of the hNgb-Cyt c complex predicted from docking, we performed all-atom MD simulations in explicit solvent.

**Figure 1:**
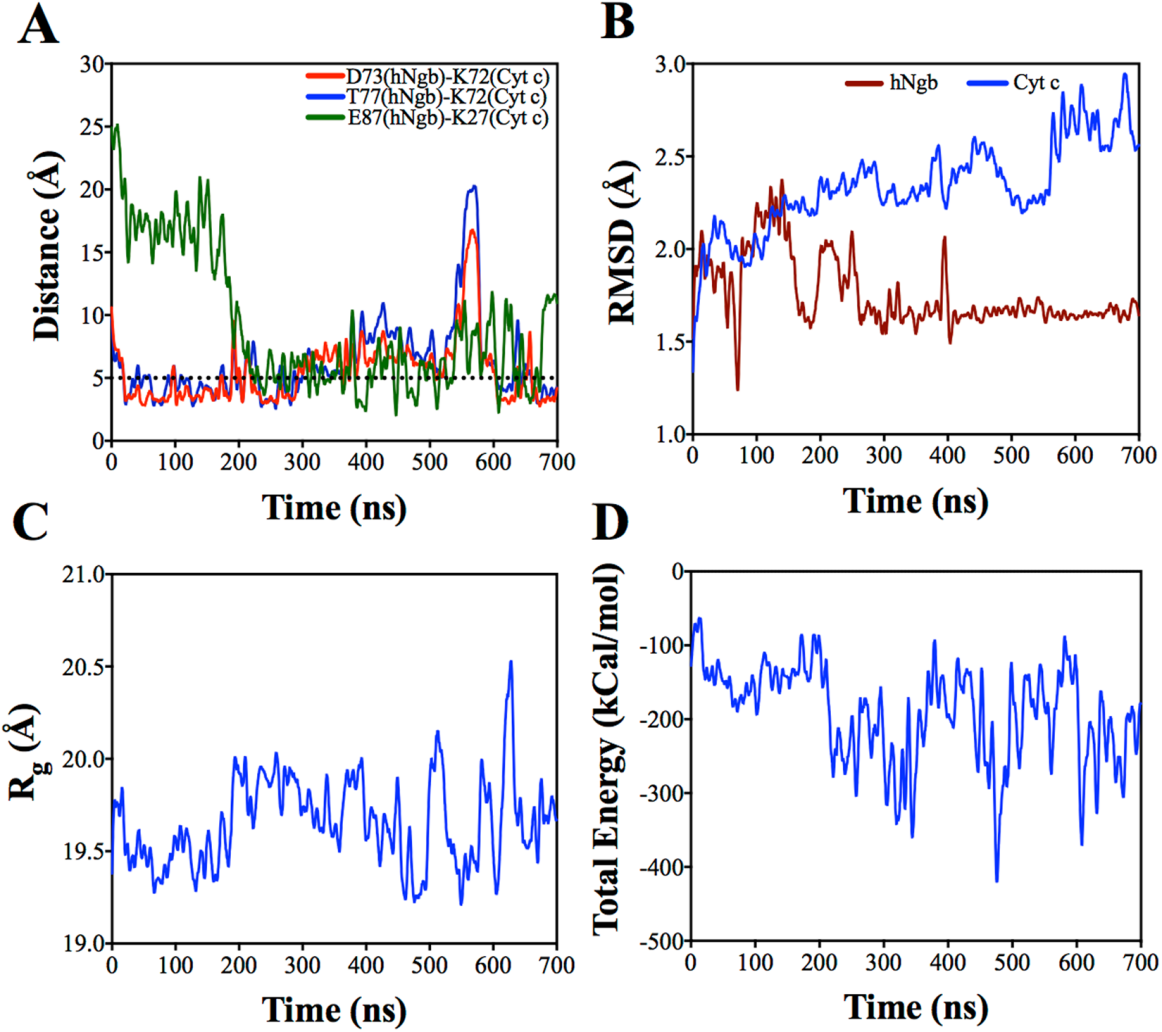
**(A)** Distance between the predicted amino acid residues that form the complex between hNgb and Cyt c. The dotted line is drawn to show a reference line at 5 Å for salt bridge. **(B)** RMSD measurements for hNgb and Cyt c. (**C**) Radius of gyration (R_g_) and **(D)** total interaction energy of hNgb-Cyt c complex as a function of simulation time.

We investigated the amino acid residue contacts between hNgb and Cyt c during complex formation. As shown in Table 1, several residues exhibit interfacial contacts. We observed two salt bridges between D73 (hNgb) and K72 (Cyt c) and between E87 (hNgb) and K27 (Cyt c) as well as a hydrogen bond between T77 (hNgb) and K72 (Cyt c). The salt bridge formed between D73 (hNgb) and K72 (Cyt c) is stable as indicated by the stable distance <5 Å^29^ shown in Fig. 1A. However, compared to the salt bridge between D73 (hNgb) and K72 (Cyt c), the hydrogen bond between T77 (hNgb) and K72 (Cyt c) is not as stable. We compared the bond stability by the number of frames that showed interaction, based on contact analysis (Materials and Methods section). For the hydrogen bonding this number is ~1200. However, for the salt bridge the number of frames are ~1700. The residues D73 (hNgb) and T77 (hNgb) are both located in the E-helix of hNgb^30^. The repositioning of the E-helix, as an exogenous ligand binds to hNgb, has been proposed to alter the affinity of hNgb to Cyt c^12^.

**Table 1:** Amino acid residues in both hNgb and Cyt c that showed interfacial contacts.

hNgb residues	Cyt c residues
E87, Y88, S91	Q16
E87	K27
S84	T28
L70, D73, T77	K72
T77, N78	K79
K67, L70, V71, A74, L85, Y88, HEME	I81
L70	F82
K67, L70	A83
L85, Y88	HEME

Several residues in one protein that establish interfacial contact with another protein are separated by coma on the same rows.

As shown in Fig. 1B, as revealed by RMSD measurements, there are large rearrangements in Cyt c structure while hNgb structure remains mainly conserved. We have also measured radius of gyration (R_g_) of the structure during the NVT simulations. As shown in Fig. 1C, R_g_ does not change significantly throughout the simulation, which suggests that the overall complex is thermally stable. Figure 1D shows the total interaction energy as a function of time. We did not observe significant fluctuation in the total energy of the system, which further assures the stability of the hNgb-Cyt c complex.

**Figure 2:**
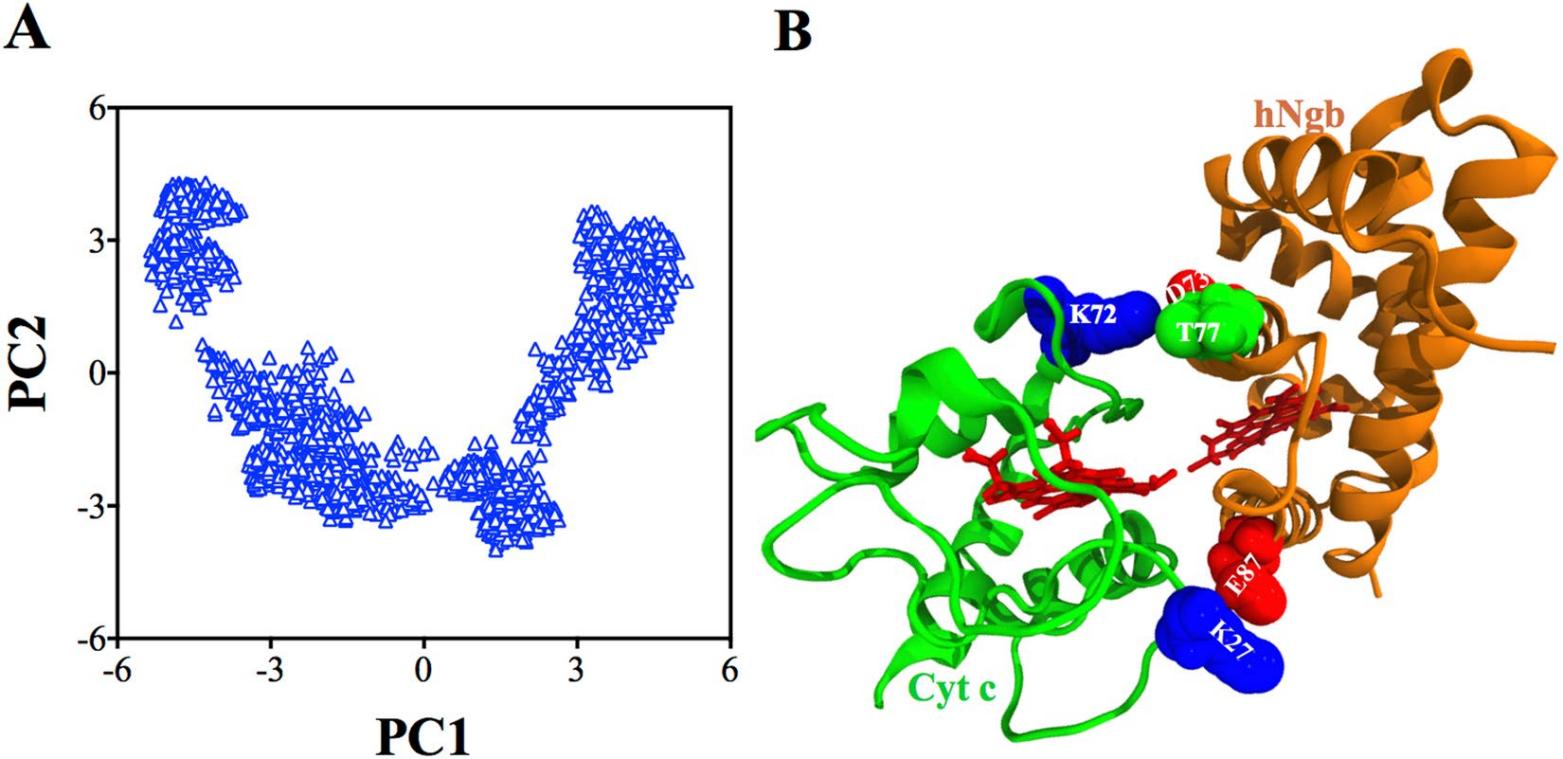
(**A**) Projection of the hNgb-Cyt c complex structures along the direction of the first two principal components (PC1 and PC2). (**B**) An averaged hNgb-Cyt c complex structure obtained from PCA based cluster analysis.

We performed principal component analysis (PCA) of the 700 ns MD simulation trajectories to evaluate the conformational differences between the hNgb-Cyt c complex structures. As shown in Fig. 2A, as compared to PC2, PC1 varies more broadly. An average hNgb-Cyt c complex structure obtained from the PCA-based cluster analysis is shown in Fig. 2B. The important residue pairs that are responsible to form specific salt bridges and hydrogen bonding are highlighted in Fig. 2B (VDW representation).

### SPR experiments confirm the involvement of the amino acid residues predicted by the MD simulation

**Figure 3:**
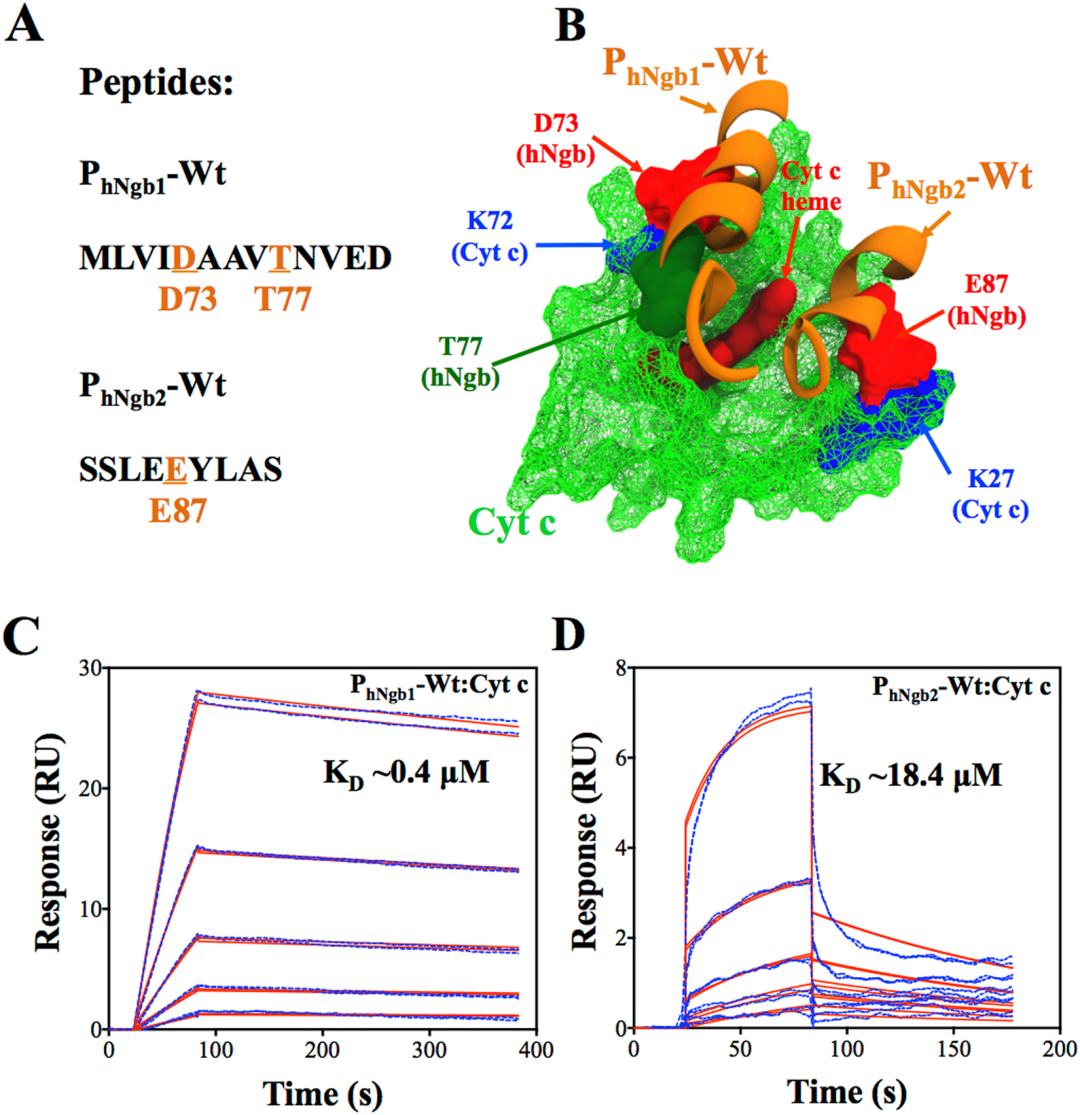
**(A)** Names and sequences of the peptides used in the SPR experiments. The amino acid residues predicted by the MD simulations to be involved in the formation of the hNgb-Cyt c complex are shown in orange (with their position in hNgb) and are underlined. **(B)** Pictorial view of sections of hNgb (orange) represented by the sequences of the peptides bound to Cyt c (green). The SPR sensorgrams (blue dashed lines) of the binding of the peptides P_hNgb1_-Wt **(C)** and P_hNgb2_-Wt **(D)**. Continuous red lines are fit to a 1:1 kinetics binding model. P_hNgb1_-Wt concentrations injected during SPR experiments were 0.625 µM, 1.25 µM, 2.5 µM, 5 µM, and 10 µM and P_hNgb2_-Wt concentrations were 6.25 µM, 12.5 µM, 25 µM, 50 µM, and 100 µM.

We performed SPR experiments to confirm the involvement of the amino acid residues predicted by the MD simulations. We used two peptides, P_hNgb1_-Wt and P_hNgb2_-Wt in the SPR experiments. The sequence of P_hNgb1_-Wt comprises of hNgb amino acids ranging from M69 to D81 and that of P_hNgb2_-Wt from S83 to S91. The peptides were dissolved in 100% DMSO and further diluted in the running buffer (10 mM HEPES pH 7.4, 150 mM NaCl, and 0.05% v/v surfactant P20 supplemented at 1% v/v DMSO) and injected over the Cyt c-immobilized surface at various concentrations. The name assignments and sequences of the peptides, used as analytes in the SPR experiments, are shown in Fig. 3A. Figure 3A displays the amino acid residues (orange color and underlined) predicted by the MD simulations to be involved in the formation of the hNgb-Cyt c complex. Figure 3B shows a pictorial view of sections of hNgb (orange) represented by the sequences of the wild-type peptides that are bound to Cyt c (green) and the amino acid residues responsible to form the complex are highlighted. Figures 3C,D show the SPR sensorgrams (colored dashed lines) of peptides P_hNgb1_-Wt and P_hNgb2_-Wt binding to Cyt c, respectively.

Qualitatively, the binding of the wild-type peptides to Cyt c confirm the prediction from the simulations that the amino acid residues mentioned above are essential for the hNgb-Cyt c complex formation. Quantitatively, we fit the SPR sensorgrams (red continuous lines), as shown in Fig. 3C, using a 1:1 kinetics model to derive the equilibrium dissociation constant (affinity or K_D_ value). A K_D_ value of 0.4 ± 0.2 μM (mean ± s.d. from three different experiments) was obtained for P_hNgb1_-Wt binding to Cyt c. We were interested in comparing affinities of the peptides P_hNgb1_-Wt and P_hNgb2_-Wt binding to Cyt c. Therefore, using the same fitting procedures, we fit the sensorgrams shown in Fig. 3D, and a K_D_ value of 18.4 ± 4.3 μM (mean ± s.d. from three different experiments) was obtained for P_hNgb2_-Wt binding to Cyt c. P_hNgb1_-Wt had a 45-fold higher affinity (lower K_D_ value) than P_hNgb2_-Wt, which is highly consistent with the stronger binding of the hNgb region represented by P_hNgb1_-Wt than that of the region represented by P_hNgb2_-Wt due to the formation of the bonds shown in Fig. 1A.

**Figure 4:**
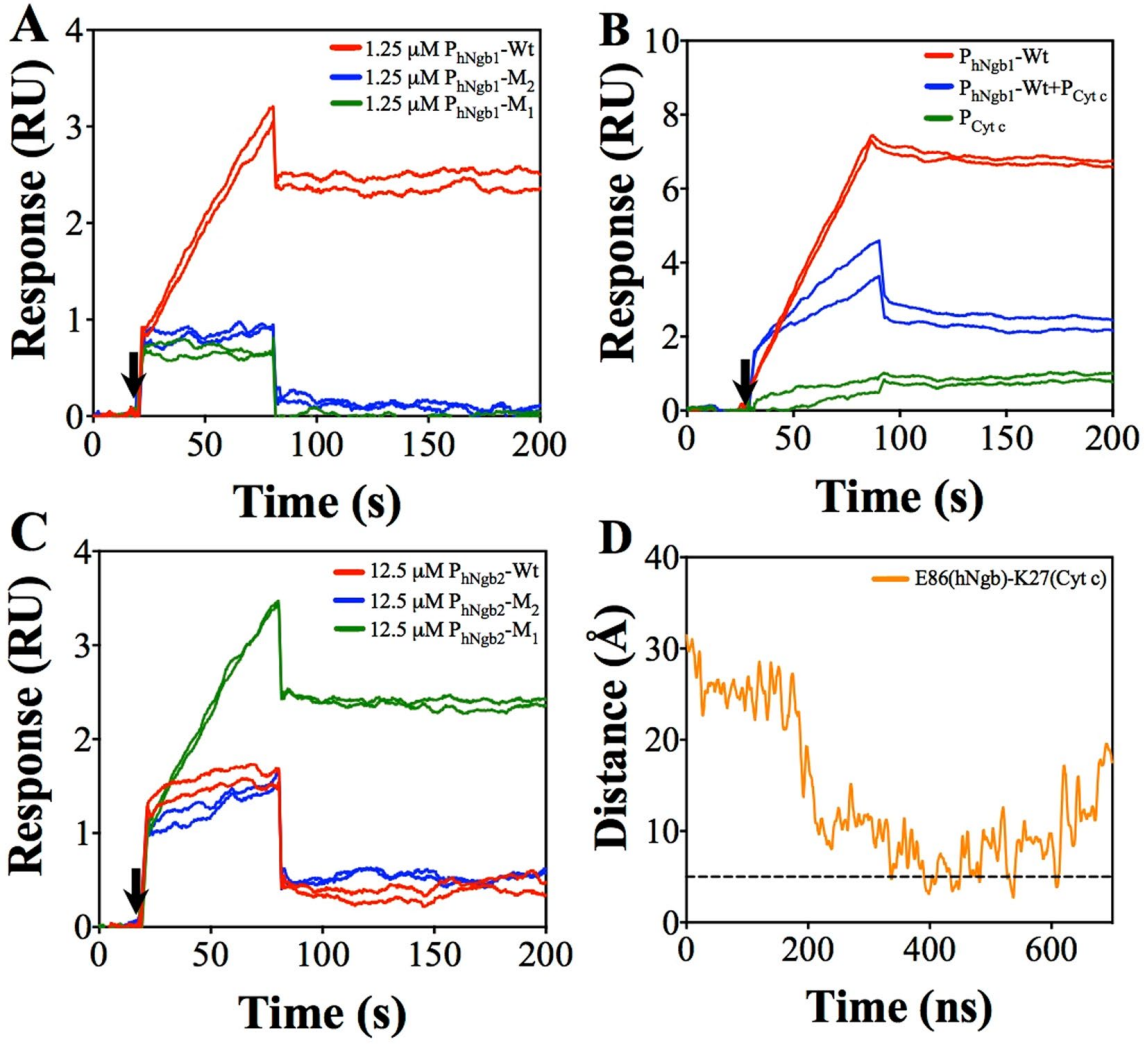
**(A)** SPR sensorgrams for qualitative comparison of bindings for P_hNgb1_-Wt, P_hNgb1_-M_1_, and P_hNgb1_-M_2_. **(B)** SPR sensorgrams for inhibition of P_hNgb1_-Wt binding by P_Cyt c_. **(C)** SPR sensorgrams for qualitative comparison of bindings for P_hNgb2_-Wt, P_hNgb2_-M_1_, and P_hNgb2_-M_2_. Black arrows in Fig. A to Fig. C show starting time of analyte injections. All peptides were injected for 60 s. **(D)** Distance between E86 (hNgb) and K27 (Cyt c) residues. The dotted line is drawn to show a reference line at 5 Å for salt bridge.

We conducted SPR experiments with mutated peptides. Qualitatively, as shown in Fig. 4A, the two mutant peptides P_hNgb1_-M_1_ (with D73A substitution) and P_hNgb1_-M_2_ (with T77A substitution) bind to Cyt c. Moreover, binding of these individual mutants showed weaker SPR signals as compared to SPR signals for P_hNgb1_-Wt, which is expected. These results also show the involvement of D73 (hNgb) and T77 (hNgb) together take part in binding to K72 (Cyt c), making a strong bonding as predicted by simulations. We could not perform quantitative investigations (via determination of K_D_ values) for the mutated peptides due to solubility issues at higher concentrations. In addition, we also conducted an inhibition experiment with a peptide P_Cyt c_ (with amino acids L68 to P76 in Cyt c) and P_hNgb1_-Wt. As shown in Fig. 4B, a mixture of P_hNgb1_-Wt and P_Cyt c_ (in 1:2 molar ratio) showed diminished signal as compared to P_hNgb1_-Wt signal. This is because P_Cyt c_ with K72 (Cyt c) interacts with P_hNgb1_-Wt via D73 (hNgb)-K72 (Cyt c) or T77 (hNgb)-K72 (Cyt c) interactions in solution and reduces the available binding sites in P_hNgb1_-Wt to bind to immobilized Cyt c onto the ship surface. P_Cyt c_, part of Cyt c sequence, does not bind to immobilized Cyt c. This experiment further confirms the involvement of the predicted amino acid residues (K72, D73 and T77) in the hNgb-Cyt c complex formation. We could not obtain purified peptide with double mutation (MLVIAAAVANVED) due to more hydrophobic in nature.

We also performed similar qualitative experiments for E87 (hNgb)-K27 (Cyt c) binding region. In addition to binding of P_hNgb2_-Wt (with E87 in hNgb), unlike weaker signals for P_hNgb1_-M_1_ and P_hNgb1_-M_2_ bindings as compared to P_hNgb1_-Wt bindings, a mutant peptide P_hNgb2_-M_1_ (with E87A substitution) also showed a stronger binding (Fig. 4C). Notably, there is E86 (hNgb) in the P_hNgb2_-Wt sequence. Based on this observation, in the absence of E87 (hNgb), we speculate that E86 (hNgb) also likely interacts with K27 (Cyt c). This possibility is also predicted in course of simulation after 300 ns, which is represented by bond distance as shown in Fig. 4D (occasionally less than 5 Å). However, the chance for this interaction is quite less as compared to the E87 (hNgb)-K27 (hNgb) interaction as shown in Fig. 1A. This observation is also supported by results from contact analysis. Based on our contact analysis, there were only ~450 frames showing E86 (hNgb)-K27 (Cyt c) interactions as compared to ~1700 frames showing E87 (hNgb)-K27 (Cyt c) interactions. We again conducted experiments with another mutant of P_hNgb2_-Wt, P_hNgb2_-M_2_ (with E86A and E87A substitutions). There is still a comparable binding between P_hNgb2_-M_2_ and P_hNgb2_-Wt. In simulation results, there is no indication of formation specific salt bridge and hydrogen bonding (except for E86 and E87) for this hNgb region. However, several interfacial contacts are observed as shown in Table 1, for this region, which might be non-specific. We speculate that this hNgb region also interacts with Cyt c via non-specific interactions like hydrophobic interactions. Therefore, we did not perform further inhibition experiments for this peptide sequence. Further extensive experiments are required for the interaction of this hNgb region with Cyt c.

### Simulation results confirm that redox reaction is likely not required to abate the apoptotic cascade

Our simulation results show that K72 (Cyt c) is one of the amino acid residues that form complex with hNgb. Importantly, this K72 residue takes part in interactions of Cyt c with apoptotic protease-activation factor-1^31^. Our simulation results show that the hNgb-Cyt c complex forms, involving K72 (Cyt c), even when both proteins in oxidized form; thus requires no redox reaction between hNgb and Cyt c for this process. Moreover, hNgb-Cyt c association is enough to suppress caspase-9 activation^19^. Hence, Cyt c likely competes with apoptosome formation by interacting with hNgb^14,31^. Therefore, our results confirm that the hNgb-Cyt c redox reaction is likely not required to abate the apoptotic cascade.

### Alignment of heme groups and structural rearrangements were observed during the stabilization of the hNgb-Cyt c complex

**Figure 5:**
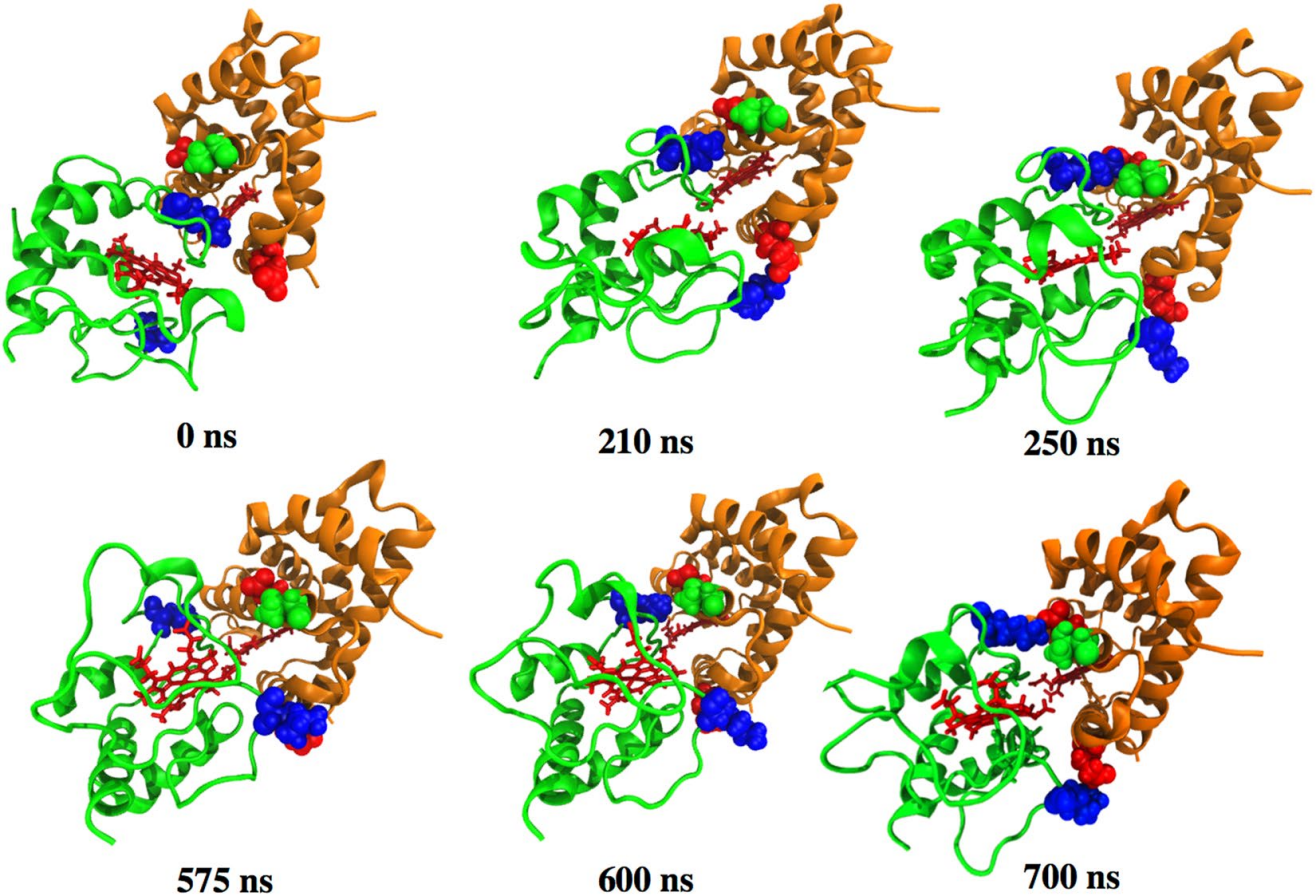
Structures of the hNgb-Cyt c complex at different times during the simulation trajectory.

Figure 5 shows the hNgb-Cyt c complex structures at the following different simulation times: 0 ns (the start of the NVT simulations), 210 ns, 250 ns, 575 ns, 600 ns and 700 ns. In the starting complex (Fig. 5 at 0 ns), the heme groups of the two proteins are oriented in different directions or planes. Interestingly, both heme groups continuously align towards the same plane during the stabilization of the hNgb-Cyt c complex as shown in Fig. 5.

There is a large rearrangement in the Cyt c structure while hNgb structure is mainly conserved as revealed by the RMSD measurements. Interestingly, at 210 ns, Cyt c rotated around an axis passing through the Fe atoms (at the center of the heme groups) of both hNgb and Cyt c. This structural rearrangement at 210 ns during the complex stabilization initiates the formation of another salt bridge between E87 (hNgb) and K27 (Cyt c) (Fig. 1A). This structural rearrangement as well as new salt bridge formation is also shown in the simulation movie (Supporting file Movie-S1.mp4, after 150 ns of the NVT simulation). In course of simulation, at around 575 ns, another major structural rearrangement was observed, which temporarily increased bond distance between D73 (hNgb) and K72 (Cyt c) as well as between T77 (hNgb) and K72 (Cyt c), as shown in Fig. 1A. At this time no major destabilization in the heme groups was observed. This temporary destabilization is due to a structural modification in Cyt c while the structure of hNgb remains conserved, as supported by RMSD measurements (Fig. 1B). This structural rearrangement is also shown in the simulation movie (Supporting file Movie-S2.mp4, after 500 ns of the NVT simulation).

## Discussion

We performed an MD simulations-based investigation of the formation and stabilization of the complex between hNgb and Cyt c. We obtained the best predicted hNgb-Cyt c complex from docking as described in the Materials and Methods section. In both models of the docked complex as proposed in a previous publication^12^, the protein interfaces involved in forming the complex are the same, with only a little difference in the initial amino acid contacts. Since MD incorporates conformational flexibility and structural adjustments (e.g. rotation of Cyt c at 210 ns as explained above) and allows optimization of the interfacial contacts of the complex, either model would give an optimized complex upon relaxation with MD. In the docking, we chose PDB ID 1OCD for Cyt c structure, with ~88% sequence homology with human Cyt c, as our template because this protein is easily available for purchase at lower cost for numerous experiments.

As supported by SPR results, our simulation results predict that the two residues D73 (hNgb) and T77 (hNgb), both E-helix residues, interact with K72 (Cyt c) and that E87 (hNgb) interacts with K27 (Cyt c) to establish the hNgb-Cyt c complex. The alignment of the heme groups as well as the interactions between the E-helix residues in hNgb and K72 in Cyt c are also crucial for the formation and stabilization of the hNgb-Cyt c complex. As previously mentioned, the repositioning of the E-helix alters the affinity of hNgb to Cyt c. This alignment in the heme groups also confirms the likely interaction between the heme groups for the formation (and stabilization) of the hNgb-Cyt c complex, which is consistent with a previous report based on docking results^14^. In addition, the involvement of K72 (Cyt c) in the formation of the complex between hNgb and Cyt c suggests that the interaction with hNgb likely competes with apoptosome formation, without requiring redox reaction between Ngb and Cyt c. Altogether, in the absence of a crystal structure, our results provide unique insight into the optimized structure of the hNgb-Cyt c complex, which has a biological role in preventing apoptosis. To the best of our knowledge, this is the first computational investigation of the molecular mechanism underlying the Ngb-Cyt c complex formation, allowing conformational flexibility and structural rearrangements. In future, following this investigation, more extensive computation-based investigations can be done, including much longer and repeated simulations for the wild type complex and all complexes with the mutation of all amino acid residues predicted in this report. Moreover, our current investigations explore avenues to conduct extensive experiments for the bindings (both specific and non-specific) of hNgb region represented by P_hNgb2_-Wt as well as calculation of quantitative binding energies for wild type and mutant proteins that could help further confirm the importance of each residue in the hNgb-Cyt c complex formation.

## Materials and Methods

### Materials

Equine heart Cyt c and dimethyl sulfoxide (DMSO) were purchased from Sigma Aldrich. The amine coupling kit, Series S sensor chip CM5, and HBS-P+ buffer (0.1 M HEPES, 1.5 M NaCl, and 0.5% v/v surfactant P20) were purchased from GE Healthcare. The peptides P_hNgb1_-Wt (sequence: MLVIDAAVTNVED), P_hNgb1_-M_1_ (sequence: MLVIDAAVANVED), P_hNgb1_-M_2_ (sequence: MLVIAAAVTNVED), P_hNgb2_-Wt (sequence: SSLEEYLAS), P_hNgb2_-M_1_ (sequence: SSLEAYLAS), P_hNgb2_-M_2_ (sequence: SSLAAYLAS), and P_Cyt c_ (sequence: LENPKKYIP), were purchased from GenScript.

### Protein structures and docking

PDB ID 4MPM (Chain B)^30^ was used for hNgb, and PDB ID 1OCD^32^ was used for Cyt c. The ZDOCK protein docking server^33^ was used to predict the hNgb-Cyt c complexes. During the docking submission, SER84 (hNgb)-PRO76 (Cyt c), ASP73 (hNgb)-LYS73 (Cyt c), and heme propionate (hNgb)-LYS72 (Cyt c) group pairs were selected as the contacting residues. Of the top 10 predicted complexes obtained from docking, only one complex with the lowest Fe-Fe distance (16.85 Å), was selected as the best predicted complex. These contacting amino acid residues and Fe-Fe distance were selected based on a previous publication^12^.

### Molecular dynamics (MD) simulations

The best predicted hNgb-Cyt c complex was used in the MD simulations. The input files (.psf and.pdb) for the complex were generated by Visual Molecular Dynamics (VMD)^34^ using the topology and parameters suggested by a previous publication^35^ for the oxidized Cyt c heme together with the CHARMM 36 force field. The complex was then solvated in a cubic box with TIP 3P (or TIP3) water and electrically neutralized by adding four Cl^−^ ions, which resulted in a cubic solvation box with dimensions of 90 × 90 × 90 Å^3^ containing 68,749 atoms. All-atom MD simulations were performed using the NAMD simulation package^36^. The MD simulations of the complex began with 10,000 steps of energy minimization using the conjugate gradient and line search algorithm. The Particle Mesh Ewald (PME)^37^ method was used for the long-range interactions with a 12 Å non-bonded cut-off. The system was then equilibrated with a 1 fs integration time step at 300 K (27 °C) for 100 ps with the protein heavy atoms harmonically restrained. After equilibration, the simulation was continued for a 100 ps NPT run with the fully unrestrained protein using a 2 fs time step. The production simulation was then propagated for 700 ns using Langevin dynamics with a damping constant of 1 ps^−1^ under NVT conditions and a time step of 2 fs.

### Surface plasmon resonance (SPR)

The SPR experiments were performed using a Biacore T200 SPR instrument (GE Healthcare). Cyt c, which was used as a ligand, was immobilized onto the CM5 chip up to a level of ~9000 Response Units (RU) in the presence of 10 mM sodium acetate buffer at pH 5.5. A standard amine coupling chemistry according to the manufacturer’s recommended protocol (GE Healthcare) was used to immobilize Cyt c. HBS-P+ buffer was diluted 10X to 10 mM HEPES pH 7.4, 150 mM NaCl, and 0.05% v/v surfactant P20 (HBS-P) in ddH_2_O and filtered through a 0.22 μM polystyrene membrane filter. HBS-P was used as the immobilization running buffer. The peptides were used as analytes to inject over the Cyt c-immobilized CM5 chip surface in various concentrations in an HBS-P+1% DMSO buffer. HBS-P+1% DMSO was used as the running buffer in the analyte-ligand binding experiments. A phosphoric acid solution (1:250 v/v ratio, H_3_PO_4_:ddH_2_O) was used to regenerate the CM5 chip sensor surface for P_hNgb1_-Wt and related mutants bindings to Cyt c, and 1 M NaCl was used to regenerate the chip for the bindings of P_hNgb2_-Wt and related mutants to Cyt c. All analytes were injected in duplicate in each cycle. The kinetics experiments were repeated for three times. All qualitative experiments, including inhibition experiments were repeated at least two times with the wild type peptides injected as positive controls in each experiment.

### Data analysis

VMD^34^ was used to analyze and visualize the structure and simulation trajectories. The total interaction energy was calculated using NAMD energy plugin available in VMD. Each frame in the NVT simulation trajectory was saved at every 200 ps. An atomic distance of 3.5 Å and angle cutoff of 30 degrees were used to trace hydrogen bonding and the same atomic distance of 3.5 Å was used as the cut off to analyze interfacial contacts and salt bridges. For contacts analysis, only residues that show contacts for more than 1000 frames were considered. Carma^38^ was used to determine the radius of gyration (R_g_) and to perform PCA as well as PCA-based cluster analyses. GraphPad prism (GraphPad Software, Inc.) was used to generate the graphs. The bonding distances between D73 (in hNgb, atom OD2) and K72 (in Cyt c, atom NZ), T77 (in hNgb, atom OG1) and K72 (in Cyt c, atom NZ), E87 (in hNgb, atom OE2) and K27 (in Cyt c, atom NZ) as well as between E86 (in hNgb, atom OE2) and K27 (in Cyt c, atom NZ) were plotted. All graphs obtained from analyses of MD simulation trajectories were averaged with 25 neighboring points and 4^th^ order smoothing polynomial using the GraphPad prism software. Biacore T200 evaluation software version 1.0 (GE Healthcare) was used to analyze the SPR sensorgrams.

## Electronic supplementary material

Supporting file Movie-S1

Supporting file Movie-S2
